# Investigating Social Ecological Contributors to Diabetes within Hispanics in an Underserved U.S.-Mexico Border Community

**DOI:** 10.3390/ijerph10083217

**Published:** 2013-07-31

**Authors:** Jean Chang, Mignonne C. Guy, Cecilia Rosales, Jill G. de Zapien, Lisa K. Staten, Maria L. Fernandez, Scott C. Carvajal

**Affiliations:** 1 Mel and Enid Zuckerman College of Public Health, Arizona Prevention Research Center, University of Arizona, 1295 N. Martin Ave, Tucson, AZ 85724, USA; E-Mails: jean0131@email.arizona.edu (J.C.); crosales@email.arizona.edu (C.R.); dezapien@email.arizona.edu (J.G.Z.); chachaml@hotmail.com (M.L.F.); 2 Mayo Clinic Arizona, Department of Health Sciences Research, 13400 E. Shea Blvd, Scottsdale, AZ 85259, USA; E-Mail: Guy.Mignonne@mayo.edu; 3 Richard M. Fairbanks School of Public Health at IUPUI, Indiana University, 714 N. Senate Avenue EF 250, Indianapolis, IN 46202, USA; E-Mail: lkstaten@iupui.edu

**Keywords:** diabetes, hispanics, U.S.-Mexico border, obesity, underserved, socio-ecological model, health disparities, health behaviors

## Abstract

Hispanics bear a disproportionate burden of diabetes in the United States, yet relations of structural, socio-cultural and behavioral factors linked to diabetes are not fully understood across all of their communities. The current study examines disparities and factors associated with diabetes in adult Hispanics of Mexican-descent (N = 648) participating in a population survey of an underserved rural U.S.-Mexico border community. The overall rate of diabetes prevalence rate in the sample, based on self-report and a glucose testing, was 21%; much higher than rates reported for U.S. adults overall, for all Hispanic adults, or for Mexican American adults specifically. Acculturation markers and social determinants of health indicators were only significantly related to diabetes in models not accounting for age. Older age, greater BMI (>30), greater waist-to-hip ratio as well as lower fruit and vegetable consumption were significantly related to increased likelihood of diabetes when all structural, cultural, behavioral, and biological factors were considered. Models with sets of behavioral factors and biological factors each significantly improved explanation of diabetes relative to prior social ecological theory-guided models. The findings show a critical need for diabetes prevention efforts in this community and suggest that health promotion efforts should particularly focus on increasing fruit and vegetable consumption.

## 1. Introduction

The World Health Organization (WHO) reports diabetes as the ninth leading cause of death worldwide and a global epidemic affecting 347 million people [1]. In the U.S., there are an estimated 25.8 million people, or 8.3% of the population, with diabetes, approximately one-third of which are undiagnosed [2]. Recent studies have projected that by 2050, the incident cases of diabetes are expected to nearly double, and the prevalence will increase to between 21% and 33% of the U.S. population [3]. Diabetes is the seventh leading cause of death in the U.S. and current trends suggest that this widening epidemic will continue and subsequent diabetes-related mortality will increase over time [2].

Although diabetes affects all groups in the U.S., non-White racial and ethnic populations suffer a disproportionate burden of the disease, associated complications, and death [2,4]. In Hispanics, the largest racial and ethnic minority group, rates of diabetes are almost twice that of Non-Hispanic-Whites [2] (*Note*: the terms Hispanic and Latino are used interchangeably and now both are included in U.S. government official designations of ethnic self-identity. These terms refer to people whose origins are of Spanish-speaking origin or ancestry). In the largest sub-group of Hispanics, Mexican Americans, the rate is further elevated [5]. This is not surprising as Mexico, like the U.S., also has one of the highest rates of obesity in the World [6] and rising obesity and diabetes rates are recognized as primary public health threats.

The prevalence of age-adjusted diagnosed diabetes in Mexican American adults in the U.S. is 13.3%, nearly double that of Non-Hispanic Whites (7.1%) [7]. Compared to a national age-adjusted average of 8.2 per 1,000 persons, rates of incident diabetes nationwide are highest among Hispanic men (13.2), Hispanic women (13.1), persons with less than high school education (15.1), those in poverty (11.2), and those who are disabled (14.9) [8]. The high rate of Type-II diabetes in Hispanic adults has been documented to contribute to substantive negative impacts on quality of life in addition to premature mortality (e.g., the 5th overall leading cause of death; mortality rates due to diabetes are 60% higher than in non-Hispanic Whites) [9,10]. In fact, diabetes mortality and morbidities represent Latino health disparities that are some of the strongest contraindications to the generally well supported Hispanic/Latino Epidemiological Paradox–where despite the presence of many traditional structural risk factors for disease such as being disproportionately of low income and low educational attainment, their overall health outcomes are comparatively favorable [11].

Along the U.S.-Mexico border, diabetes rates in Hispanics have been further reported to be double those in other parts of the USA [12,13,14]. Additionally, Mexican Americans living in rural and unincorporated areas experience a further disproportionate burden of diabetes and associated comorbidities when compared to Non-Hispanic Whites, or to Mexican Americans residing in other regions of the USA [15,16,17]. Additionally, a recent study showed that obesity rates within the largest U.S.-Mexico border state were highest in areas with high Hispanic concentration and low rates of higher education [18].

While there are various ongoing research efforts with Hispanic and Mexican American populations addressing factors related to obesity and diabetes, such as in the Hispanic Community Health Study [19], there is a need for more current data on factors using representative surveys of rural [20] as well as border-residing Hispanic communities. There is consistent evidence for excess rates of obesity and diabetes in Hispanics residing along the U.S.-Mexico border, however, there is less evidence concerning which factors are most linked to the prevalence of diabetes in this region—such knowledge is critical to the development and effective targeting of health promotion efforts.

The present study sought to use a socio-ecological framework [21,22] to identify and model factors related to diabetes in Hispanics residing in a border community. The current study was conducted as part of a large, representative household survey recently conducted in Douglas, Cochise County, Arizona. Aspects of this locale are important to understanding the context and implications of diabetes there; this locale is federally recognized as rural [23], medically underserved, and has a documented shortage of primary care, mental and dental health professionals [24].

Our social ecological approach to diabetes requires consideration of contextual influences, such as social determinants (structural factors) and cultural factors, as well as behavioral and biological factors [20,21,22]. Acculturation, generally expressed as exposure and adaptation to a new culture, is one factor of focus in this study. Acculturation is recognized as an important socio-cultural variable related to Hispanic health, and for illuminating health disparities within diverse ethnic groups that make up the Hispanic population [25]. However, while greater acculturation is often related to higher prevalence of diabetes in Hispanics in general [5,26,27] there are some contradictory findings with regards to Mexican-origin Hispanics [5,28,29]. To understand acculturation-related health disparities, the interpretations should also consider socio-economic and/or community background [30,31,32,33]. Consequently, the current study uses a panel of well-defined structural factors [33], in particular those relating to health relevant resources, in addition to acculturation markers, to represent important contextual influences.

Behavioral factors (behavioral reports and specific cognitions) [12,28,34] and adiposity indicators (biological determinants [22]) will also be considered as explanatory factors in this study. Guided by the larger theoretical framework, all explanatory factors will be examined individually, then jointly tested with other variables of the same social ecological level of influence. Finally, a comprehensive model will be tested with all relevant explanatory factors, with biological factors in the last modeling stage. 

## 2. Methods

### 2.1. Design, Participants and Sampling

A cross-sectional research design was conducted to explore significant explanatory factors related to diabetes in our target population. The current study’s participants were drawn from a randomized cross-sectional community survey of 708 residents that was commissioned by an academic-community partnership to collect community health needs and planning data. The survey was conducted between October 2010 and September 2011, and is representative of the municipality of Douglas, Arizona (17,387 residents) as well as the adjacent unincorporated area of Pirtleville, Arizona (1,744 residents) [35]. Census data from 2010 estimates that these municipalities have a combined total of 19,122 residents; 84% identified as Hispanic/Latino, 75% reported Spanish as the dominant language in their home, 32% reported living below the federal poverty level and 36% reported having less than a high school education) [35]. Strata of census blocks were identified by ethnicity (Hispanic/non-Hispanic) and socioeconomic status (SES) in order to ensure an accurate reflection of this locale’s socio-demographic distribution. Occupied housing units were randomly chosen from the selected census areas.

Resident community health workers (“promotoras”/CHWs) who were experienced interviewers were enlisted to recruit participants and collect all data. These promotoras received training on the project protocol, survey administration, and if needed a refresher course on clinical measurement of height, weight, and blood glucose. The promotoras visited each household to verify occupation and to explain the purpose and scope of the study. Study objectives were explained in both English and Spanish and informed consent was obtained by promotoras (all fully bilingual). All present residents over the age of 18 of the randomly selected households were eligible and invited to participate. Upon consent, measurements were conducted in participants’ homes. There was no monetary incentive for participation in the survey. The participation rate was 86% (805 households were reached and 112 had potential participants refuse). Only those participants who self-identified as being of Hispanic origin or descent (Mexican, Mexican American, Latino/a, Latin American or Chicano/a) were included in the current study (N = 648).

### 2.2. Measures

Explanatory variables included objectively measured anthropometric estimates as well as self-report survey questionnaire data collected by the promotoras. Questionnaire data included acculturation measures and other socio-demographic information (age, sex, marital status, education, and insurance). Language acculturation was measured with two English language proficiency Likert-format items: one about understanding spoken English and one on speaking English. These items were highly associated (r = 0.99; α = 0.99) and thus summed (Mean, SD, range = 5.52, 2.71, 2–8). They were part of four items selected from a well-validated orthogonal acculturation scale [36], though the Spanish proficiency items were not used in further analysis due to ceiling effects—only three of the respondents indicated “poor” or “very-poor” on either item. Length of residency and nativity questions completed the acculturation descriptors [36].

Reports on smoking history, alcohol consumption, physical activity, diet, and beliefs about diabetes were assessed, which all relate to individual level influences within a social ecological perspective. Obesity status was represented by body mass index (BMI) and waist-to-hip ratio (WHR) recorded by the interviewers. BMI was calculated using weight (kg)/(height (m))^2^ derived from medical grade scales and stadiometers. For analysis and table presentation, the following BMI categories were used: 18–24.9; 25–29.9; 30 and above. Waist-to-hip Ratio was categorized by >0.99 for men and >0.92 for women based on the optimal cardiovascular risk identification criteria for Mexican American adults [37]. Current/former smoking (*vs*. never smoking) was measured by a “yes” or “no” response to the question of “Have you smoked more than 100 cigarettes or five packs of cigarettes in your life?” [38]. Alcohol consumption was measured by number of drinks per year then categorized based on Dietary Guidelines for Americans for “moderate” (or less) drinking as consuming up to two drinks per day for men and up to one drink per day for women [39]. Vigorous/moderate exercise was determined from whether respondents met the CDC’s guidelines in their responses to the brief version of the International Physical Activity Questionnaire [40]: 150 min of moderate-intensity exercise per week or 75 min of vigorous-intensity exercise per week [41]. Inactivity (marker of sedentary status) was determined by whether respondents reported 6 h or more of sitting per day [42]. Diet was assessed by the fruit and vegetable consumption component of the CDC-Behavioral Risk Factor Surveillance System (BRFSS). While the CDC recommends a minimum serving of three vegetables and two fruits per day [43], the 2009 BRFSS shows that people who consume more than five fruits and vegetable per day represent 23% nationwide [44] and in our study the estimate was a mere 3% of the population. Thus, in our study we use the criteria of whether respondents ate three or more servings of fruits and vegetables. Respondents were also asked to report “yes” or “no” to whether they believed that diabetes was preventable (Table 1).

**Table 1:** Descriptive characteristics of sample and the explanatory variables (N = 648).

Variable		N (%)
*Sex*		n = 648
	Male	234 (36.1)
	Female	414 (63.9)
*Marital Status*		n = 648
	Married/consensual union	382 (59.0)
*Education (in years)*		n = 648
	No School	5 (0.8)
	1–8	179 (27.6)
	9–11	106 (16.4)
	12-GED	172 (26.5)
	Some College	139 (21.5)
	College or more	47 (7.3)
*Insurance Status*		n = 648
	Medicaid	276 (42.6)
	Medicare	170 (26.2)
	Private	80 (12.3)
*U.S. born*		n = 646
	Yes	247 (38.1)
	No	399 (61.6)
*Residency in the U.S. (in years)*		n = 646
	>18	518(80.0)
	≤18	128(20.0)
*English Language Acculturation Score*		n = 648
	<5	280 (43.0)
	≥5	368 (57.0)
*Smoking (prior or current smoker)*		n = 648
	Yes	195 (30.1)
	No	453 (69.9)
*Moderate or Heavy Alcohol Consumption*		n = 648
	Yes	95 (15.0)
	No	553 (85.0)
*Physical Exercise (Vigorous or Moderate)*		n = 648
	Yes	127 (20.0 **)**
	No	521 (80.0)
*Inactivity (6 or more hours)*		n = 646
	Yes	28 (4.3)
	No	618 (95.7)
*Fruits and Vegetable Consumption (3 or more)*		n = 646
	Yes	208 (32.0)
	No	438 (68.0)
*Is Diabetes Preventable?*		n = 648
	Yes	443 (68.4)
	No	205 (31.6)
*BMI*		n = 648
	18–24.9	145 (22.4)
	25–29.9	240 (37.0)
	30 and Above	263 (40.6)
*Waist-to-Hip Ratio (WHR)*		n = 642
	>0.92 for women, > 0.99 or greater for men	469 (73.1)
	≤0.92 for women, ≤ 0.99 or greater for men	173 (26.9)

Participants were classified as having diabetes or an abnormal glucose test if: (1) they reported that a doctor had previously told them they had diabetes (excluding gestational diabetes) or high blood sugar; or (2) they presented a fasting glucose level of 126 mg/dL or higher during screening; or (3) they presented a random glucose level of 200 mg/dL or higher [45]. Blood glucose levels were measured using capillary draw with the OneTouch^®^ system (LifeScan Inc, Milpitas, CA, USA), a high performing screening glucometer [46]. Participants were asked whether they had fasted for at least 8 h before the blood glucose measure. If the response was no, the reading was considered a random measure.

### 2.3. Analysis

Logistic regression was used to determine significant explanatory variables of diabetes. First, univariate logistic regressions were performed. Next, logistic regression models with age as a covariate were used to determine associations of each factor statistically controlling for age. Finally, hierarchical logistic regression models were tested to determine how age and sets of socio-cultural, biological and behavioral factors relate to the prevalence of diabetes [22]. Hosmer-Lemeshow tests were performed to determine goodness of fit for the hierarchical logistic regression models. The *p*-value criteria used for evaluating statistical significance in the univariate and hierarchical logistic regressions was 0.05 (Table 2). We applied a *p*-value criteria of 0.25 for inclusion of all specific explanatory factors from the age-adjusted models into the subsequent theory-based multivariate hierarchical logistic regressions tested [47]. Sample power to detect small and modest differences in diabetes attributable to our model variables was also evaluated. With a sample of 648, two-tailed significance level of 0.05, and expected diabetes prevalence around 20%, we would have over 85% power to detect a clinically significant 5% rate difference and 99% chance to detect a rate difference of 7% [48].

**Table 2:** Univariate logistic regression models predicting diabetes (N = 648).

Variable		Univariate Crude Odds		Univariate Age-adjusted	
		COR	95% CI	AOR	95% CI
*Demographics*					
	Age	**1.05**	**1.04–1.07** ***	–	–
	Gender (male)	0.97	0.65–1.43	0.92	0.60-1.41
*Structural Factors*					
	Medicaid (yes)	1.36	0.93–1.98	1.45	0.97–2.19 ^MV^
	Medicare (yes)	**3.16**	**2.13–4.71** ***	1.70	0.95-3.03 ^MV^
	Private Insurance (no)	1.62	0.85–3.08	1.25	0.63–2.49
	Education ^Ͻ^	**2.58**	**1.75–3.81** ***	1.32	0.85–2.03 ^MV^
	Marital Status (married)	1.05	0.72–1.54	1.06	0.70–1.60
*Cultural Factors*					
	Birth Place (Mexico)	**2.11**	**1.38–3.22** ***	1.58	0.97–2.43 ^MV^
	Residency in U.S. (≥18 years)	**1.49**	**0.89–2.48** ***	1.09	0.63–1.90
	English Acculturation Score (≤5)	**2.22**	**1.52–3.26** ***	1.45	0.96–2.20 ^MV^
*Behavioral Factors*					
	Smoking ^Ω^	1.47	0.80–2.71	1.23	0.64–2.36
	Alcohol Consumption^ ξ^	**0.30**	**0.14–0.63** **	**0.40**	**0.18–0.88** *
	Vigorous/Moderate exercise ^¶^	**1.62**	**1.11–2.36** *	1.12	0.74–1.70
	Fruit and Vegetable Consumption ^ɸ^	**1.85**	**1.19–2.86** **	**2.46**	**1.52–3.97** ***
	Inactivity (sitting) ^∞^	1.50	0.65–3.49	1.61	0.63–4.16
	Diabetes beliefs ^π^	**1.72**	**1.17–2.54** **	1.29	0.84–1.96 ^MV^
*Biological Factors*					
	BMI 25-29.99 ^µ^	1.36	0.80–2.33	1.44	0.80–2.60 ^MV^
	BMI 30 and above ^µ^	1.59	0.94-2.68	**2.21**	**1.23–3.98** **
	Waist-to-Hip Ratio (WHR) ^ϒ^	**2.40**	**1.62–3.58** ***	**2.01**	**1.30–3.09** **

Note: Diabetes was determined from positive self-report of clinical diagnosis or elevated serum blood glucose. COR = Crude Odds Ratio. AOR =Adjusted Odds Ratio. CI = Confidence Interval. * *p* < 0.05; ** *p* < 0.01; *** *p* < 0.001; MV Indicates *p* < 0.25 (for inclusion in multivariable models, [49]). References for referents: ^Ͻ^: High school or higher level of education; ^Ω^ <100 cigarettes or five packs in life; ^ξ^ <2 drinks per day for men and < 1 drink per day for women; ^¶^ <150 min of moderate-intensity exercise per week and <75 min of vigorous-intensity exercise per week; ^ɸ^ <3 vegetables and fruit per day; ∞ < 6 h; π Belief that diabetes is preventable; µ BMI 18–24.99; ^ϒ^ >0.99 for men and >0.92 for women.

## 3. Results

In the sample, 138 respondents (21.3%) met one of the two diabetes criteria for the prevalence calculation; 133 (20.5%) reported a diagnosis; five cases had elevated glucose estimates but did not indicate a prior diagnosis (48 persons with self-reported diabetes also had elevated glucose estimates). The mean age of respondents was 52.06 (SD ± 18.81). Using the age categories and sampling weights of the most recent National Health Interview Survey (1997–2011) [49], the age-adjusted prevalence of self-reported diabetes was 15.1% in this sample. 

Table 1 presents demographic and frequencies of the study participants; 64% were women (n = 414), more than 70% of participants had no college educational experience or degree, 43% were Medicaid beneficiaries, 59% were married, 62% were born outside of the U.S., and 80% had been in the U.S. 18 years or longer. Regarding behavioral and biological factors, about 70% of the sample reported as having never smoked, with equal distributions (15%) of current and former smokers; 15% reported moderate/heavy drinking. While less than 5% reported high inactivity, only 20% engaged in moderate or vigorous exercise. About one-third (32%) of respondents consumed three or more fruits and vegetables per day. Nearly 70% of respondents said diabetes is generally preventable. The mean BMI was nearly 30 (Mean, SD = 29.55, 6.39). Approximately 40% were within the obese range in BMI and 70% had elevated waist-to-hip (WHR) ratios. 

Significant factors were initially identified using univariate logistic regressions producing crude odds ratios and test statistics (Table 2). These results show that participants were more likely to have diabetes if they were older, had less than a high school education, had Medicare, were born in Mexico, had lived in the U.S. more than 18 years, and were less proficient in English. Regarding the behavioral and biological factors, persons were more likely to have diabetes if they: reported less than moderate alcohol consumption, fail to engage in moderate or more exercise, ate less than three servings of fruits and vegetables per day, reported that diabetes was not preventable, and have high WHR (Table 2).

Next, each specific factor was examined in a model with age as a covariate. No demographic or acculturation variable remained significantly associated with diabetes in these models (*Note*: models with length of residency in 10 year intervals were also tested with logistic regression and the results were similar to those with the previously described dichotomous coding of this explanatory variable. The latter models are presented to restrict confounding of years of residency and participants’ age, and to facilitate interpretation). When adjusted for age, two behavioral and two biological factors were significantly associated with diabetes: those reporting lower alcohol consumption, those who ate less than three servings of fruits and vegetables per day, those obese (BMI > 30), and those with high WHR were more likely to have diabetes.

Hierarchical multivariable logistic regressions were then tested that included all age-adjusted factors with probability values < 0.25, those that were not statistically significant though included in later stages of modeling are indicated as “MV” in Table 2. Results from this model are presented in Table 3. Blocks of structural factors (e.g., education, health insurance) and cultural factors (e.g., acculturation) did not significantly improve explanation of the prevalence of diabetes when age was controlled (Δχ_(3)_² = 4.57 (*p* = 0.206); Δχ_(2)_² = 1.68 (*p* = 0.452)). Adding the set of behavioral factors, however, did improve the fit of the model (Δχ_(4)_² = 20.64, *p* < 0.001). Fruit and vegetable consumption and alcohol consumption remained significant in the model with behavioral factors and age also considered. When biological factors were added as a last step in the hierarchical logistic regression, fruit and vegetable consumption remained a significant along with age, BMI (>30) and WHR. Adding the set of biological factors also significantly improved model fit (Δχ_(3)_² = 17.44, *p* = 0.001). The most robust explanatory variables were eating fewer fruits and vegetable (*p* = 0.001), age (*p* = 0.001) and higher waist-to-hip ratio (*p* = 0.003). BMI > 30 (*p* = 0.016) and lower alcohol consumption (*p* = 0.028) were also identified as significant explanatory variables relating to diabetes status controlling for age, social and cultural factors.

**Table 3:** Age-adjusted hierarchical logistic regression models predicting diabetes (N = 643).

Model	Model 1: Age χ_(1)_² = 88.81, *p*< 0.001 Nag R^2^ = 0.203		Model 2: Structural Factors χ_(4)_² = 93.38, *p*< 0.001; Nag R^2^ = 0.212		Model 3: Cultural Factors χ_(6)_² = 95.06, *p*< 0.001;Nag R^2^ = 0.216		Model 4: Behavioral Factors χ_(10)_² = 115.70, *p*< 0.001; Nag R^2^:0.258	Model 5: Biological Factors χ_(13)_² = 133.24, *p*< 0.001;Nag R^2^:0.294		
Variable	AOR	95% CI	AOR	95% CI	AOR	95% CI	AOR	95% CI	AOR	95% CI
Demographics										
Age	**1.06** ***	**1.04–1.07**	**1.07** ***	**1.05–1.09**	**1.06** ***	**1.04–1.09**	**1.07** ***	**1.04–1.09**	**1.07** ***	**1.05–1.09**
Structural Factors										
Education			0.90	0.57–1.42	1.03	0.62–1.72	1.09	0.64–1.85	1.15	0.67–1.97
Medicaid (yes)			1.31	0.85–2.03	1.27	0.82–1.97	1.28	0.81–2.01	1.32	0.84–2.10
Medicare (yes)			0.66	0.37–1.19	0.69	0.38–1.24	0.71	0.39–1.30	0.67	0.36–1.25
Cultural Factors										
Birth Place (Mexico)					1.26	0.71–2.25	1.39	0.76–2.51	1.43	0.78–2.61
Acculturation Score					0.87	0.50–1.53	0.93	0.52–1.65	0.88	0.48–1.58
Behavioral Factors										
Alcohol							0.45	0.20–1.00	0.41 *	0.18–0.94
Fruit/Veg Consumption ^ɸ^							**0.41** ***	**0.25–0.67**	**0.42** ***	**0.25–0.69**
Diabetes Knowledge							0.74	0.47–1.14	0.74	0.47–1.16
Biological Factors										
BMI25–29.99 ^µ^									1.34	0.75–2.60
BMI > 30^µ^									**2.15** *	**1.16–3.98**
Waist–to–Hip Ratio ^ϒ^									**2.04** **	**1.30–3.20**

Note: Diabetes was determined from positive self-report of clinical diagnosis or elevated serum blood glucose. Consecutively tested models included all prior sets of covariates. Nag = Nagelkerke. * *p* < 0.05 ** *p* < 0.01 *** *p* < 0.001; Reference for referents with significant associations: ^ɸ^ <3 vegetables and fruit per day, ^µ^ BMI 18–24.99, ^ϒ^ >0.99 for men and >0.92 for women.

## 4. Discussion

The current study presents data on diabetes-related factors in Hispanics, including social, cultural, behavioral, and biological influences, from a recently completed community survey. Diabetes prevalence was established both by a common marker in self-report surveillance studies as well as from elevated capillary blood glucose readings above standard screening guidelines. The prevalence of diabetes was 21% in the sample; all but 1% reflected in the self-report of a health professional’s diagnosis. In terms of total diabetic burden for Hispanics in this Southeast Arizona community, this is over 50% greater than the national rates for Hispanics or Mexican American adults [5], and higher than the 15%–18% rates from other Hispanic border samples [11,13,16]. Also, the age adjusted rate was about 25% greater than that indicated for Hispanics in the most recent national data [49]. Of further note only 7% of these Hispanic adults, drawn from this proportional and randomized household survey, had completed a college degree and about two-thirds had no insurance or had Medicaid. These findings show there remains a critical need for more diabetes prevention and treatment services for the residents of this predominantly Hispanic participating community, and based on reports from other sparsely populated U.S.-Mexico border communities [14,17,50], this is likely evident for the wider rural U.S.-Mexico border region.

Having lower education, being on Medicare, being married as well as all three acculturation markers, were significantly associated with crude odds for diabetes. However, controlling for age removed all statistically significant relationships among these contextual influences. This suggests the former variables acted as proxies for sample age differences in our sample and care should be taken when interpreting within Hispanic disparities from unadjusted diabetes prevalence estimates (most commonly reported in surveillance studies). The lack of significant cultural and resource factors in the age-adjusted models may also be due to the relative cultural homogeneity of Hispanics in the sample (e.g., very little variation in Spanish linguistic acculturation despite using items from a well-validated scale; few college graduates; few with private health insurance). These findings illustrate the importance of interpreting acculturation and resource factors within community contexts; this may be particularly true in border communities as well as others that are Hispanic enclaves (communities concentrated in Hispanic residents and where Hispanic cultural influences are pervasive).

Along with age, the other factors significantly related to diabetes in the final model were behavioral and biological ones from a social ecological perspective [22]. Body mass index, a standard measure of obesity that reflects consumed calories and ties closely to diet and physical activity, was positively associated with diabetes, but only at higher (obese) levels. Those with a BMI of 30 or above were two times more likely to have diabetes than those with a BMI less than 25. However, the association was stronger for an alternative measure of obesity, waist-to-hip (WHR) ratio. Currently there is debate about the role of BMI in Hispanic health and mortality [51], and if our findings in the context of explaining objectively measured diabetes are indicative, WHR may be a more important obesity-related chronic disease risk indicator [37].

The findings also showed that eating fewer fruits and vegetables was associated with a higher likelihood of diabetes in all models, and was along with age, the strongest explanatory variables. Of note however, very few persons ate the recommended levels of five or more fruits and vegetables, though respondents who reported eating less than three per day had over double the risk of a positive diabetes status relative to those who eat at least three per day. Our results further confirm addressing food choice as an important primary target for social ecological interventions to address diabetes [21,22]. Future studies may also consider an environmental scan in underserved border communities to examine the availability and affordability of fresh produce and more in-depth qualitative investigations about other contexts [7]. Health promotion efforts addressing community food environments may further contribute to improved dietary outcomes [20].

Low levels of physical activity and inactivity were not related to diabetes after adjustment for age or other significant covariates in our models. While we did use a well-established measure, the International Physical Activity Questionnaire, it should be noted we employed the shorter version in order to limit overall participant burden. Perhaps the long version [40], which discriminates activity in various domains (e.g., work, leisure, home) would have identified specific domains of physical activity of importance. Also, other work has highlighted the importance of considering physical activity contexts for Hispanic adults [21,31,34], and such attention may make physical activity assessments in this population stronger predictors of their diabetes and other chronic diseases.

There are other important strengths and limitations within the current study. The randomized household sampling of the parent project is a major strength. While we are limited in inferences to a specific community, we have a relatively large and representative sample to generate prevalence estimates with precision. Further, the larger study afforded measures of a broad range of factors related to diabetes, and in turn their inclusion and the analytic models construction were guided by social ecological theory. This data also well compliments other efforts to understand Hispanic health such as an intensive ongoing study in four major urban environments [52]. It is important to note that the response rate was outstanding, particularly for a study without monetary incentives. While many studies have recognized the critical role of promotoras/CHWs in health promotion delivery efforts [15,53,54], this study and others [15,50] also illustrate their effectiveness in contributing to high quality data collected for community health and needs assessment surveillance efforts.

Another important limitation of the study is the cross-sectional design. While it is less plausible that the socio-cultural factors were causally influenced by diabetes status, empirical evidence for temporal precedence or causation cannot be generated from the current data. For instance, the fact that current moderate or more drinking was associated with lower risk of diabetes (assuming the modest association observed in this study, significant at the *p* < 0.05 level but not a more stringent criteria, is replicable) may reflect behavioral changes post diagnosis and post clinical recommendations, rather than any true protective effect of this level of drinking. Due to field and related data collection constraints, we could not collect hemoglobin A1C in serum nor require fasting by participants for a clinical diagnosis of diabetes [5]. Finally, were not able to assess all aspects of a social ecological perspective on diabetes causal factors, such as family history of diabetes and genetic biomarkers that are likely associated with additional diabetes risk, nor social support and social network variables that may have protective associations [55].

## 5. Conclusions

The current study identified a high diabetes prevalence rate (over 20%) in Hispanics residing in an underserved border community. There were also important disparities in diabetes associated with socio-cultural and structural factors including acculturation markers, consistent with a social ecological perspective. However, acculturation factors were not robust when considering age and structural factors in our theory-guided models. This suggests that while they may be useful in identifying where to target health promotion resources to high diabetes prevalence groups (e.g., creating materials and diabetes-related messaging in Spanish, reaching the population through low-income community, senior or health centers), acculturation change processes whining this community do not appear to be contributing to diabetes onset. Older age, having high risk WHR, and eating few fruits and vegetables per day were associated with diabetes across all models. Thus, health promotion and surveillance efforts with border Hispanic populations should be particularly attuned to fruit and vegetable consumption as well as adiposity indicators.
